# The impact of cancer and chemotherapy during pregnancy on child neurodevelopment: A multimodal neuroimaging analysis

**DOI:** 10.1016/j.eclinm.2020.100598

**Published:** 2020-10-21

**Authors:** J. Blommaert, A. Radwan, C. Sleurs, C. Maggen, M. van Gerwen, V. Wolters, D. Christiaens, R. Peeters, P. Dupont, S. Sunaert, K. Van Calsteren, S. Deprez, F. Amant

**Affiliations:** a Department of Oncology, KU Leuven, Leuven, Belgium; b Department of Imaging & Pathology, KU Leuven, Leuven, Belgium; c Department of Gynecology, The Netherlands Cancer Institute, Antoni van Leeuwenhoek, Amsterdam, Netherlands; d Princess Máxima Center for pediatric oncology, Utrecht, Netherlands; e Centre for the Developing Brain, School of Biomedical Engineering & Imaging Sciences, King's College London, London, United Kingdom; f Department of Electrical Engineering, ESAT/PSI, KU Leuven, Leuven, Belgium; g Department of Radiology, University Hospitals Leuven, Leuven, Belgium; h Department of Neurosciences, KU Leuven, Leuven, Belgium; i Department of Gynaecology and Obstetrics, University Hospitals Leuven, Leuven, Belgium; j Department of Development and Regeneration, Unit Woman and child, KU Leuven, Leuven, Belgium; k Center for Gynaecologic Oncology Amsterdam, Netherlands Cancer Institute and University Medical Centers, Amsterdam, Netherlands

**Keywords:** Cancer in pregnancy, Chemotherapy during pregnancy, Neurodevelopment, MRI, Diffusion, Resting-state fMRI, Morphometry

## Abstract

**Background:**

This study applies multimodal MRI to investigate neurodevelopment in nine-year-old children born to cancer-complicated pregnancies.

**Methods:**

In this cohort study, children born after cancer-complicated pregnancies were recruited alongside 1:1 matched controls regarding age, sex and gestational age at birth (GA). Multimodal MRI was used to investigate whole-brain and subcortical volume, cortical structure (using surface-based morphometry), white matter microstructure (using fixel-based analysis) and functional connectivity (using resting-state blood-oxygen-level-dependant signal correlations). Graph theory probed whole-brain structural and functional organization. For each imaging outcome we conducted two group comparisons: 1) children born after cancer-complicated pregnancies versus matched controls, and 2) the subgroup of children with prenatal chemotherapy exposure versus matched controls. In both models, we used the covariate of GA and the group-by-GA interaction, using false-discovery-rate (FDR) or family-wise-error (FWE) correction for multiple comparisons. Exploratory post-hoc analyses investigated the relation between brain structure/function, neuropsychological outcome and maternal oncological/obstetrical history.

**Findings:**

Forty-two children born after cancer-complicated pregnancies were included in this study, with 30 prenatally exposed to chemotherapy. Brain organization and functional connectivity were not significantly different between groups. Both cancer and chemotherapy in pregnancy, as compared to matched controls, were associated with a lower travel depth, indicating less pronounced gyrification, in the left superior temporal gyrus (p_FDR_ ≤ 006), with post-hoc analysis indicating platinum derivatives during pregnancy as a potential risk factor (*p* = .028). Both cancer and chemotherapy in pregnancy were related to a lower fibre cross-section (FCS) and lower fibre density and cross-section (FDC) in the posterior corpus callosum and its tapetal fibres, compared to controls. Higher FDC in the chemotherapy subgroup and higher FCS in the whole study group were observed in the anterior thalamic radiations. None of the psycho-behavioural parameters correlated significantly with any of the brain differences in the study group or chemotherapy subgroup.

**Interpretation:**

Prenatal exposure to maternal cancer and its treatment might affect local grey and white matter structure, but not functional connectivity or global organization. While platinum-based therapy was identified as a potential risk factor, this was not the case for chemotherapy in general.

**Funding:**

This project has received funding from the European Union's Horizon 2020 research and innovation program (European Research council, grant no 647,047), the Foundation against cancer (Stichting tegen kanker, grant no. 2014–152) and the Research Foundation Flanders (FWO, grants no. 11B9919N, 12ZV420N)

**Research in context**

**Evidence before this study**

Only few studies have investigated neurodevelopment after prenatal exposure to maternal cancer and are most often limited in follow-up time or included children of heterogenous ages. While short-term follow-up studies, in children up to three years old, did not show any differences in cognitive outcome other than prematurity-related effects, longer term follow-up studies showed discrepancies in behaviour, executive functioning and verbal intelligence. Moreover, potential neurotoxicity of chemotherapy has been shown in preclinical research and in cancer survivor studies.

**Added value of this study**

This study is the first to apply multimodal MRI for assessing brain development in children born after cancer-complicated pregnancies. Prenatal exposure to maternal cancer and its treatment were related to altered development of local grey and white matter structure, but no alterations in functional connectivity or global organization were seen. While platinum derivates were indicated as a potential risk factor for affected grey matter development, this was not the case for chemotherapy in general.

**Implications of all the available evidence**

Long-term follow-up until adulthood in large multicenter cohorts is necessary for assessing the risks of prenatal exposure to maternal cancer and for disentangling multiple cancer and therapy-related risks. Balancing between pros and cons on the use of chemotherapy during pregnancy, the current data favour the use of chemotherapy during pregnancy when clinically indicated.

Alt-text: Unlabelled box

## 1 Introduction

Cancer complicates about one in 1000 pregnancies [1]. This diagnosis unavoidably leads to difficult medical and ethical decisions [2]. While treatment delay can worsen the maternal prognosis, starting cancer treatment during pregnancy and preterm delivery might impact foetal development. Over the last twenty years, evidence of short-term safety of cancer treatment during pregnancy has been growing [1,3], resulting in an increased number of mothers being treated during their pregnancy and less pregnancy terminations, iatrogenic preterm deliveries and treatment delays [1].

Chemotherapy is contraindicated before 12 weeks of pregnancy due to the increased risk of congenital anomalies [4]. Chemotherapy exposure in the second and third trimester of pregnancy has been associated with more growth restriction and preterm delivery [1]. Moreover, the impact of chemotherapy on neurocognitive development remains a concern as the foetal brain is rapidly developing during the second and third trimesters of pregnancy through processes of neurogenesis, neuronal migration, synaptogenesis, etc. [5,6]. One mouse study observed prenatal exposure to vinblastine and doxorubicin to affect brain development, impacting both brain structure and behaviour [7]. Such early life impact might only become apparent in later life as the child develops into adolescence [8] and adulthood [5].

To date, prenatal exposure to cancer treatment does not appear to be associated with altered neurocognitive development in children aged up to three years old [9], [10], [11], [12]. However, the associated frequent prematurity has been identified as an important risk factor for lower cognitive scores in early childhood [[9], [10], [11],^13^]. Later during childhood however, there are indications that prenatal chemotherapy exposure might be linked to more internalizing and externalizing behavioural problems [10], as well as a lower performance on verbal intelligence [14] and executive functioning tasks [15].

In multiple cancer populations, chemotherapy has been associated with altered neurocognition, as well as both structural and functional brain changes [16]. However, when assessing the neurocognitive impact of prenatal cancer treatment exposure, secondary effects such as low birth weight, prematurity and maternal malnutrition, depression, stress and anxiety might also impact neurodevelopment [17]. It has been hypothesized that the observed impact on psycho-behavioural development of the offspring, might be partially explained by the psychosocial impact of a cancer diagnosis on the mother during pregnancy [14,18,19].

State-of-the-art multimodal magnetic resonance imaging (MRI) of the brain has proven to be a valuable tool in characterizing and understanding impaired and healthy neurodevelopment. In this study, we employ state-of-the-art multimodal MRI techniques, in combination with psychological testing, as well as obstetrical and oncological parameters, to increase understanding of the potentially detrimental effects of cancer and its treatment during pregnancy on structural and functional brain development in the offspring at nine years old.

## 2 Methods

### 2.1 Participants

This cohort study prospectively included children of the Belgian cohort from the international follow-up study of the International Network on Cancer, Infertility, and Pregnancy (INCIP) [3]. Children in the study group were born to mothers with a cancer diagnosis during pregnancy. Children in the control group, born to healthy mothers, were matched on a 1:1 ratio regarding gestational age at birth (GA, maximum 1 week difference), age (9 years old) and sex. Participant recruitment is further detailed by van Gerwen et al. [20]. Exclusion criteria in both groups were major obstetrical and neonatal complications which possibly affect cognitive development (e.g. neonatal infections, pre-eclampsia), which was checked through medical records and parents-reported questionnaires on prenatal history and general health. All children were tested between 2015 and 2020 at the age of nine years, at the university hospital of Leuven, Belgium. This sample partially overlaps with the samples in our previous research [9,11,14,15], though assessments in these cohort studies were often performed at different ages and never included any MRI-derived measures.

Full scale intelligence was assessed by a psychologist, using the Wechsler Intelligence Scale for Children (WISC, version III or V) [21,22]. The WISC-IV was not used as it was never translated to dutch, with the WISC-V being introduced in 2018, replacing the WISC-III. Verbal intelligence of children was included as an outcome parameter when the WISC III was used. The Child behaviour Checklist (CBCL) was used to assess behavioural development through three scales of internalizing, externalizing and total behavioural problems [23]. The behaviour Rating Inventory of Executive Function (BRIEF) [24] was used to assess executive functioning through three composite scores: behavioural regulation, metacognition and the global executive composite score.

Data on oncological treatment, obstetrical outcome and demographics, were collected via the INCIP registry as described elsewhere [1]. Customized birth weight percentiles were calculated using the BULK GROW (v8.0.4, 2019) calculator, adjusting for nationality, maternal height/weight, parity, sex and GA [25].

The study was approved by the local ethical committee and conducted in accordance with the Declaration of Helsinki. This study is registered at ClinicalTrials.gov: NCT00330447. Informed consent was signed by one of the parents or legal guardians upon participation. The full study protocol is available at http://www.cancerinpregnancy.org/study-protocols.

### 2.2 MRI acquisition

All children underwent a half-hour whole-brain MRI scanning protocol, using the same scanner (3T Philips Achieva, 32-channel phased-array head coil). To avoid subject motion, children were familiarized with the scanner prior to entering the scanner room, were repeatedly encouraged not to move and a movie was presented during all non-functional images. Multiple MRI modalities were acquired: T2-weighted fluid-attenuated inversion recovery images (T2-FLAIR, resolution = 0.68 × 0.68 × 4 mm, 1 mm slice gap, TR/TE/TI = 9000/120/2500 ms, FA = 90°, FOV = 230 × 139 × 187 mm), high-resolution T1-weighted images (MPRAGE, resolution = .98x.98 × 1.2 mm, TR/TE = 9.6/4.6 ms, FA = 8°, FOV=160 × 256 × 256 mm), multi-shell diffusion-weighted images (b-value = 0/700/2000 s/mm² with respectively 6/30/60 uniformly distributed gradient directions, resolution = 2.5 × 2.5 × 2.5 mm, FOV = 240 × 240 × 125 mm, TR/TE = 7000/72 ms, FA = 90°, Phase encoding = AP, halfscan = 0.766, one additional b0 image with reversed phase-encoding) and resting-state functional images (rs-fMRI, using T2*-weighted Echo-planar imaging, resolution = 3.59 × 3.59 × 4 mm, FOV = 230 × 230 × 120 mm, FA = 90°, TE/TR = 33/1700 ms, acquisition time = 7 min, 250 vol + 4 initial dummy volumes).

### 2.3 Data analysis

A detailed overview of all analysis steps performed can be found in the supplementary materials. All analyses included multiple steps of bias, motion and artefact correction [26], [27], [28], [29], [30], [31], [32], as well as visual and quantitative quality assurance [32,33].

First, T2-FLAIR and T1-weighted images were evaluated by a clinical neuroradiologist for abnormalities. Second, grey matter (GM) morphometrical features were analysed using Mindboggle (v1.3.8) [29]. Total brain, GM and white matter (WM) volumes, as well as the volumes of each subcortical structure [34] were estimated. Mindboggle was used to estimate mean thickness, surface area, mean travel depth and mean curvature for each cortical region. Third, WM microstructure was investigated with a fixel-based analysis [36] on the diffusion-weighted images, resolving crossing fibre populations within a voxel, using MRtrix [35] (v3.0). This analysis gives a within-fixel measure of fibre density (FD), a macroscopic Jacobian-based measure of fibre cross-section (FCS) and a combined measure termed Fibre Density and Cross-section (FDC) [36]. Next, functional connectivity was estimated based on bivariate correlations in the rs-fMRI signal between brain regions. In order to limit the number of statistical comparisons, only regions of the default mode (DMN), fronto-parietal (FPN), dorsal attention (DAN) and salience networks (SN) were selected from the CONN toolbox (v19.b) [37] network atlas, based on the a priori hypothesis of potential impact of chemotherapy on attention and executive functioning. This resulted in a total of 17 regions included in this analysis. Finally, whole-brain structural and functional organization, using normalized weighted graphs respectively based on whole-brain constrained spherical deconvolution (CSD) tractography [38] and bivariate correlations between the rs-fMRI timeseries, were investigated using graph theory measures of characteristic path length, global and local efficiency and clustering coefficient [39].

### 2.4 Statistical analysis

Demographic and psycho-behavioural parameters were compared between study and control groups (SPSS v.19.0), using Mann-Whitney U-tests.

For each imaging outcome parameter two group analyses were conducted: 1) All children born after cancer-complicated pregnancies (the whole study group) versus matched controls and 2) the subgroup of children with prenatal chemotherapy exposure versus matched controls. Based on previous observations of the impact of prematurity on the neurocognitive outcome in children born after cancer-complicated pregnancies [[9], [10], [11],^13^], a general linear model (GLM) with group, normalized GA and the group-by-GA interaction was used. When no significant main effect of GA or group-by-GA interaction was observed, this model was simplified to only include group as a predictor. Intracranial volume (ICV) was added as a covariate in the analysis of FCS, FDC [40] and measures of volume. False-discovery-rate (FDR) correction was used for multiple comparisons in ROI-to-ROI functional connectivity and grey matter structure, whereas family-wise-error (FWE) correction in combination with connectivity-based fixel enhancement (CFE) [41] was used for the fixel-based analyses, as further detailed in the supplementary materials. Significance was assessed at *p* <  .05.

Mean values of parameters in regions with a significant group effect were extracted for each participant. Exploratory post-hoc analyses were performed with SPSS investigating the association between these regional parameters of brain functioning/structure with psycho-behavioural functioning and obstetrical/oncological outcomes, using Spearman correlations for numerical variables and Mann-Whitney *U test* for categorical variables. The following obstetrical and oncological variables were used for post-hoc analyses in the whole study group: mother deceased (yes/no), chemotherapy during pregnancy (yes/no), radiotherapy during pregnancy (yes/no), surgery during pregnancy (yes/no), and customized birth weight percentile [25]. Within the group with chemotherapy during pregnancy, the following clinical variables were additionally tested: anthracyclines (yes/no), platinum derivatives (yes/no), 5-FU (yes/no), cyclophosphamide (yes/no), GA at start chemotherapy and duration of chemotherapy (adjusted for chemo regimen) during pregnancy.

### 2.5 Role of funding

The funding sources had no role in writing of the manuscript or the decision to submit it for publication.

## 3 Results

### 3.1 Sample size and demographics

**Fig. 1:**
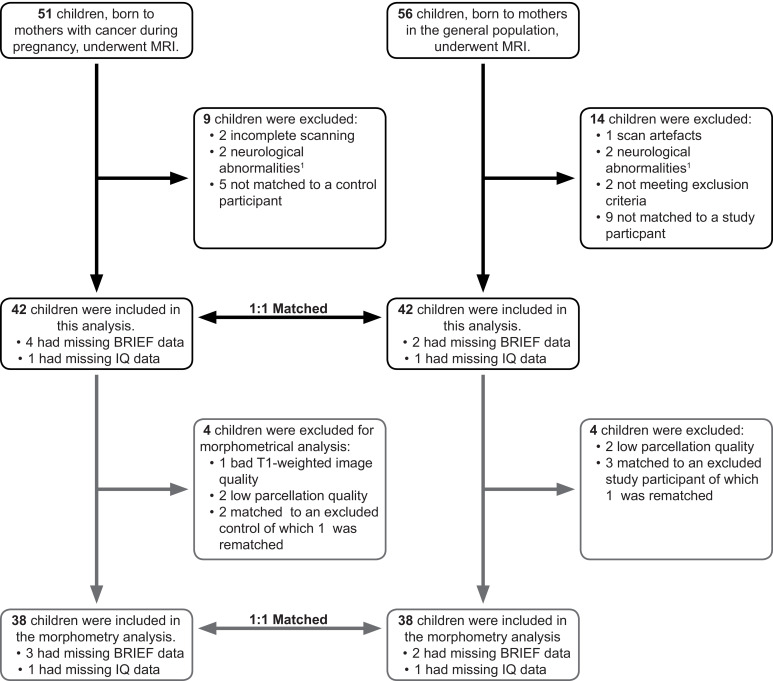
Flow diagram of recruitment for this study. Of the 51 study and 56 control participants who were scanned, 42 of each group were included in the final analysis. See supplementary materials for an overview of observed neurological abnormalities.

In total, 84 children (42 in each group) were included in the final analysis, of whom 6 (3 in each group) were excluded for the morphometrical analysis (Fig. 1). From the initial dataset, 4 children, of whom two were prenatally exposed to chemotherapy and the other two being control children, were excluded due to incidental neurological findings, detailed in the supplementary materials. For none of the cases, a direct link could be made to the maternal cancer history or treatment.

Population characteristics, clinical history and neuropsychological outcomes are shown in Table 1. Thirty mothers in the study group (71%) received chemotherapy during pregnancy, of which five (16%) additionally received radiotherapy and 24 (80%) underwent surgery. Chemotherapy during pregnancy varied in regimen (see Table 1:B), GA at start therapy (Median = 20 weeks 4 days, interquartile range = 18 weeks 2 days to 25 weeks 5 days, minimum = 14 weeks) and duration (Median =  12 weeks 2 days, interquartile range = 9 weeks 7 days to 16 weeks 1 day). Eleven mothers (26%) did not receive any systemic treatment during pregnancy, of whom 5 women did undergo surgery during pregnancy. One mother received Trastuzumab only during pregnancy. Breast cancer was the most often diagnosed cancer (*n* = 25, 60%), followed by haematological (*n* = 5, 12%) and gynaecological (*n* = 4, 10%) cancers. Within the study group, intelligence testing was performed using WISC-III in 30 children and using WISC-V in 11 children.

**Table 1:** demographics and clinical history A: Population characteristics. Prematurity classification is reported in accordance to the WHO classification: Very preterm children have GA 28-32 weeks, moderate to late preterm children have GA 32-37 weeks. Ethnicity was determined by the self-reported ethnicity of both parents. Between group differences were assessed using Mann-Whitney *U test*. *Ethnicity was compared as Caucasian vs. non-caucasian. Q1: first quartile. Q3: third quartile. GA: gestational age at birth. LGA: large for gestational age, defined as above the 90^th^ percentile. SGA: small for gestational age, defined as below the 10^th^ percentile.

	Study group (n = 42)			Control group (n = 42)			
	Median	Q1	Q3	Median	Q1	Q3	*p-value*
**Age (years)**	9.19	9.07	9.27	9.34	9.12	9.67	***.008***
**GA (weeks+days)**	36 + 3	34 + 4	37 + 7	36 + 1	34 + 3	38+0	*.941*
**Birth weight (g)**	2648	2099	3013	2930	2268	3213	*.365*
**Maternal age at birth (years)**	32	30	35	31	29	33	***.036***
	Count	Percentage		Count	Percentage		
**Prematurity**							*.958*
Very preterm	3	7%		2	5%		
Moderate to late preterm	23	55%		24	57%		
Full term	16	38%		16	38%		
**Birth weight percentile**							
SGA	7	17%					
LGA	2	5%					
**Twins**	6	14%		1	2%		*.109*
**Sex (n male)**	21	50%		21	50%		*1*
**Ethnicity**							*1
Caucasian	38	90%		37	88%		
African	4	10%		0	0%		
Mixed	0	0%		5	12%		
**Level of education parents**							
**Mother**							*.541*
Primary school	2	5%		0	0%		
Secondary school	11	26%		6	14%		
Bachelor	12	29%		21	50%		
Master	17	40%		15	36%		
**Father**							
Primary school	2	5%		0	0%		*.605*
Secondary school	16	38%		15	36%		
Bachelor	9	21%		12	29%		
Master	15	36%		15	36%		
**Smoking during pregnancy**	3	7%		2	5%		*.676*
**Drugs during pregnancy**	0	0%		0	0%		*1*
**Alcohol during pregnancy**	3	7%		5	12%		*.712*
**Mother deceased**	6	14%		0	0%		***.026***

**Table 1B:** **Maternal disease and treatment during pregnancy.** 5-FU: 5-Fluorouracil. FEC: 5-fluorouracil, epirubicin and cyclophosphamide. FAC: 5-fluorouracil, doxorubicin (adiamycin) and cyclophosphhamide. AC: doxorubicin and cyclophosphamide. EC: epirubicin and cyclophosphamide. ABVD: Doxorubicin, Bleomycin, Vinblastine, Dacarbazine.

Maternal disease (n=42)	Count	%
Breast cancer	25	60%
Cervical cancer	3	7%
Ovarian cancer	1	2%
Hodgkin Lymphoma	3	7%
Tongue cancer	3	7%
Leukaemia	2	5%
Brain tumour	2	5%
Melanoma	1	2%
Kidney carcinoma	1	2%
Colon cancer	1	2%
**Maternal treatment**		
**during pregnancy (n=42)**		
Chemotherapy	30	71%
Targeted therapy (Trastuzumab)	1	2%
Radiotherapy	5	12%
Surgery	29	69%
No treatment	6	14%
**Chemotherapy regimen (n=30)**		
FEC/FAC	11	37%
AC/EC	7	23%
ABVD	3	10%
Cisplatin	3	10%
Carboplatin and 5-FU	2	7%
5-FU	1	3%
Daunorubicin/Cytarabine	1	3%
Epirubicin	1	3%
Temozolomide	1	3%

**Table 1C:** **Neuropsychological outcomes of the child.** Between group differences were assessed using Mann-Whitney *U test* WISC: Wechsler Intelligence Scale for Children (version III or V). CBCL: Child Behavior Checklist. BRIEF: Behavior Rating Inventory of Executive Function. n_s_: number of children in the study group. n_c_: number of children in the control group. Q1: first quartile. Q3: third quartile.

	Study group (n=42)			Control group (n=42)			
	Median	Q1	Q3	Median	Q1	Q3	*p-value*
**WISC**							
Full scale intelligence	106	96	114	109	102	119	***.028***
Verbal intelligence (WISC-III, n_s_=30,n_c_=29)	108	95	114	111	103	117	*.285*
**CBCL (T-scores)**							
Internalizing problems	53	45	61	48	43	58	*.303*
Externalizing problems	49	41	54	44	40	51	*.170*
Total problems	52	44	61	47	43	53	*.111*
**BRIEF (T-scores)**							
Behavioral regulation	50	43	60	45	42	54	*.357*
Metacognition	52	45	60	52	47	58	*.700*
Global executive composite score	51	43	61	51	44	57	*.451*

52 children (62%), 26 in each group, were born preterm (before 37 weeks GA), of which the majority was born late preterm (GA = 32–37 weeks, study group *n* = 23, control group *n* = 22). In the study group, 17% of children were born small for gestational age (SGA, defined as below 10^th^ customized birth weight percentile). Unfortunately, six children (14%) in the study group had lost their mother by the time of assessment, whereas all mothers in the control group were alive. Mann-Whitney *U test* revealed a small but significant difference in age between both groups (study group: median = 9.19 years, interquartile range = 9.07–9.27 years, control group: median = 9.34 years, interquartile range = 9.12–9.67 years, *p* = .008). Mothers in the study group were on average older at birth compared to the control group (study group: median = 32 years, interquartile range = 30–35 years, control group: median = 31 years, interquartile range = 29–33 years, *p* = .036). For both groups, all psycho-behavioural measures were within normal ranges, though children in the study group showed a slightly lower total IQ (median = 106, interquartile range = 96–114,*p* = .028), compared to controls (median = 109, interquartile range = 102–119).

### 3.2 Imaging analysis

No significant differences were found in total brain (*p* = .14), WM (*p* = .30) or GM (*p* = .09) volume (Supplementary Table 2). Significantly lower cortical travel depth in the left superior temporal cortex (Table 2), indicating less pronounced gyrification, was observed in both the study group (p_FDR_ = 0.002,F(1,74) = 19.75) and the chemotherapy subgroup (p_FDR_ = 0.006,F(1,53) = 17.9), compared to matched controls. In both analyses, GA at birth was associated negatively with mean curvature, indicating a more outward-curving surface, in the left posterior cingulate cortex (resp. p_FDR_ = 0.03,F(1,72) = 13.16 and p_FDR_ = 0.03,F(1,50) = 12.6).

**Table 2:** Cortical morphometric measures. Only measures with a significant effect of group, GA or group by GA are shown. Significance is assessed at *p* < .05, FDR (Benjamini-Hochberg) corrected for assessing 62 regions.

Study group	Parameter	Region	Study group		Control group		P_FDR_		
			Mean	SD	Mean	SD	Group	GA	Group by GA
All cancers (*n* = 38)	Travel depth	Left superior temporal	8.25	0.60	8.83	0.54	*.002*	*.535*	*.966*
All cancers (*n* = 38)	Mean curvature	Left posterior cingulate	-3.41	0.25	-3.48	0.23	*.72*	*.033*	*.857*
Chemo (*n* = 27)	Travel depth	Left superior temporal	8.21	0.63	8.86	0.50	*.006*	*.535*	*.966*
Chemo (*n* = 27)	Mean curvature	Left posterior cingulate	-3.42	0.26	-3.47	0.26	*.99*	*.032*	*.857*

**Fig. 2:**
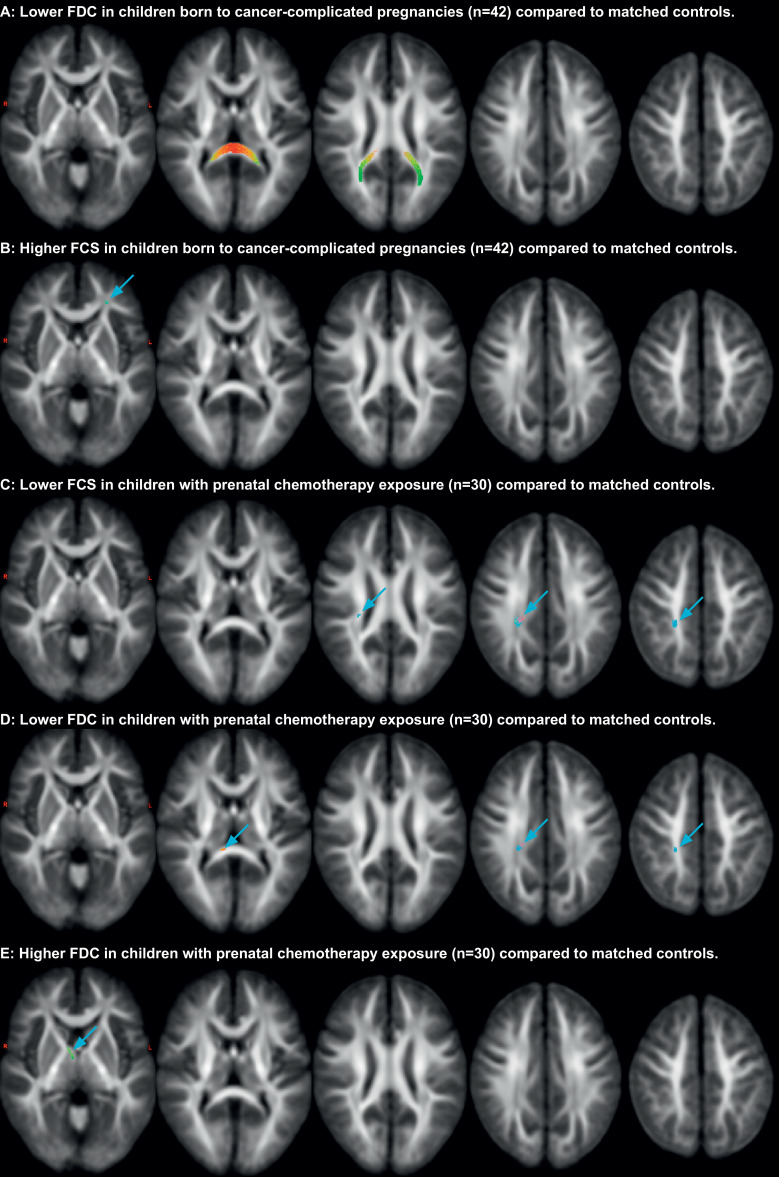
Observed differences in WM microstructure. Significance is assessed at *p* < .05, using connectivity-based fixel enhancement [41] with family-wise-error correction. All results are rendered with 200,000 streamlines (as described earlier [36]), mapped on 10 mm interval slices and coloured with conventional directional colour encoding. FCS: fibre cross-section. FDC: Fibre density and cross-section.

Concerning WM microstructure, a significantly lower FDC (Fig. 2:A ,7–17% mean difference, 465 fixels) was observed in study group children compared to controls bilaterally in the forceps major of the corpus callosum (CC) including its occipital tapetal fibres and a higher FCS (Fig. 2:B ,4–5% mean difference, 8 fixels) in the subcortical white matter of the left dorsolateral prefrontal cortex region. The latter fixels most likely belong to the anterior thalamic radiation, though the inferior fronto-occipital fasciculus or uncinate fasciculus are also possibilities as all these tracts traverse this region. Compared to controls, children with prenatal chemotherapy exposure showed a lower FCS (Fig. 2:C ,6–10% mean difference, 112 fixels) in the right tapetal fibres towards the post-central sulcus, a lower FDC (Fig. 2:D ,11–22% mean difference, 29 fixels) in the right tapetal fibres in the centrum semiovale deep to the post-central gyrus and a small group of fixels in the right side of the splenium of the CC, and a higher FDC (Fig. 2:E ,13–23% mean difference, 11 fixels) in the right anterior thalamic radiation. No significant effects in any fixel-based measures of GA or its interaction with maternal cancer or chemotherapy during pregnancy were found. Abovementioned findings are visualized at a trend level (p_FWE_ < 0.1) in Supplementary fig. 1.

No significant effects of cancer/chemotherapy during pregnancy, GA or their interaction in ROI-to-ROI functional coherence were observed after correction for multiple comparisons. However, when applying a more liberal multiple comparison correction for the ROI-to-ROI functional connectivity, accounting for the 17 regions (instead of the total number of connections) using a family-wise error correction, a significantly lower connectivity between the right lateral pole and intraparietal sulcus is observed in both the whole study group (p_FWE,ROI_ = 0.026,*T* = -3.32) and the chemotherapy subgroup (p_FWE,ROI_ = 0.050,*T* = -3.15), compared to controls. Finally, no significant effects were observed in the structural or functional whole-brain graph measures.

### 3.3 Relationship to maternal clinical history and psychological outcome

**Fig. 3:**
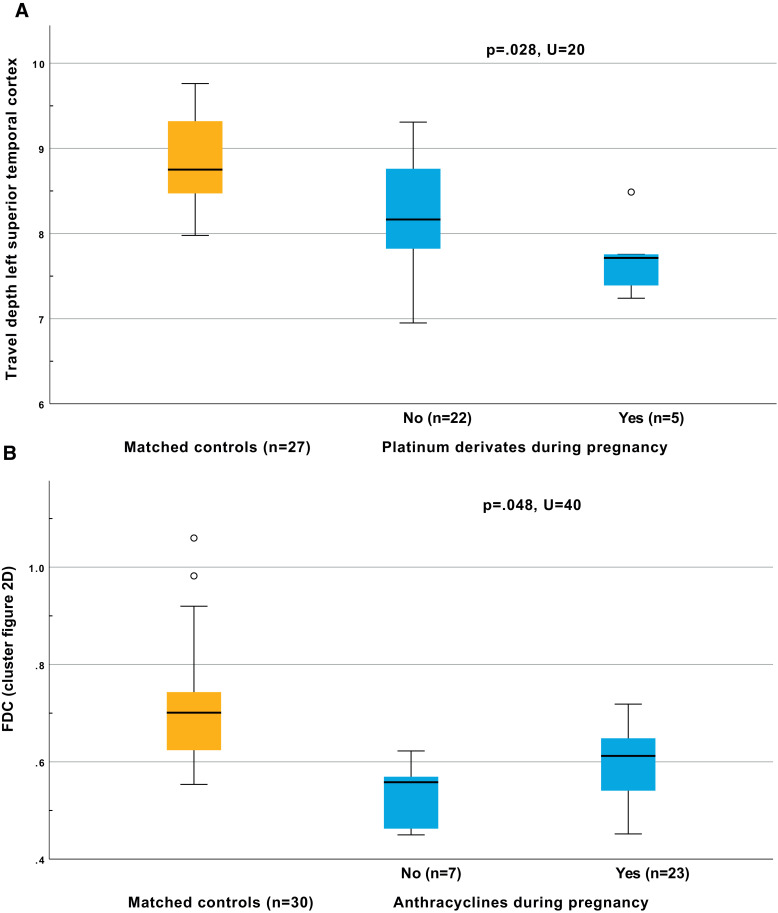
Relationship of brain differences with maternal clinical history. Mann-Whitney *U test* was used to assess significant relations (*p* < .05). Boxplots indicate minimal, maximal, quartile and median values. Outliers (indicated by circles) are defined as outside the 1.5 interquartile range.

None of the psycho-behavioural or maternal clinical parameters, were significantly related to any of the brain differences in the whole study group. While chemotherapy was never indicated as a significant risk factor, it did always trend towards more extensive impact. Within the subgroup of children with prenatal chemotherapy exposure, Mann-Whitney *U test* revealed significantly lower travel depth in the left superior temporal cortex in children exposed to platinum derived chemotherapy during pregnancy compared to other chemotherapies (*p* = .028,*U* = 20,Fig. 3:A). In the regions where we observed significantly lower FDC in prenatally chemotherapy exposed children compared to matched controls (Fig. 2:D), the FDC was less reduced for anthracyclines, compared to other chemotherapeutic agents (*p* = .048,*U* = 40, Fig. 3:B).

## 4 Discussion

To our knowledge, this is the first study applying multimodal MRI to assess brain structure and function in children born of cancer-complicated pregnancies. We found macro- and microstructural differences within the WM and GM between children born to women with cancer compared to matched controls, but no significant differences in functional connectivity or whole-brain organization were observed. Furthermore, no correlations between local structural brain differences and psychological outcome were noted. This indicates that these local structural effects have a limited effect on global and functional organization of the brain.

The differences observed in this study cannot be solely ascribed to the effect of chemotherapy, but rather to a combination of secondary mechanisms. Indeed, although differences in WM microstructure resemble results in literature of cancer survivors receiving intravenous chemotherapy [42,43], chemotherapy during pregnancy was not identified as a significant risk factor in any of the post-hoc analyses. The psychosocial impact of a cancer diagnosis during pregnancy might partially explain these findings. A recent study identified maternal death as a potential risk factor in their findings of decreased verbal intelligence in children born after cancer-complicated pregnancies [14] and another study described a relationship between infant behavioural functioning and maternal psychological wellbeing [19]. However, given that only 12/42 mothers in the study group did not receive chemotherapy during pregnancy and the heterogeneity of chemotherapy regimens used, we cannot exclude that some chemotherapeutics might directly or indirectly affect neurodevelopment.

In the whole study group, as well as in the subgroup of prenatally chemotherapy-exposed children, compared to their respective matched controls, we found a lower cortical travel depth in the left superior temporal gyrus, indicating an impact on the gyrification of the cortex. Gyrification of the brain has been found to peak around 30 weeks of gestation [44], with the left superior temporal gyrus developing slightly later than its right counterpart [45]. The left superior temporal cortex has an established role in the language network [46], which might link current findings of decreased gyrification in this region to earlier findings of lower verbal intelligence [14]. However, a direct link between travel depth in this region and verbal intelligence was not observed here.

Post-hoc exploratory analyses indicated platinum derivatives as a potential risk factor for this decreased gyrification. Platinum derivatives have a high transplacental transfer [47], [48], [49] and have earlier been associated with a higher risk for SGA [1]. Moreover, 3/5 platinum cases received cisplatin which has been associated with potential hearing loss [14,[50], [51], [52]], potentially impacting language development. However, as no data on hearing functionality was available in this cohort, a direct link could not be established.

Regarding the WM, we observed a lower FDC in the splenium of the CC and its tapetal fibres in the whole study group and a significantly lower FDC and FCS in the chemotherapy subgroup in the same region, suggesting a regional thinner axonal bundle diameter and lower axonal count. Similarly to this study, diffusion-weighted imaging has indicated impaired WM microstructure in the posterior part of the CC in survivors of paediatric soft tissue and bone sarcomas [42], as well as in adult breast cancer survivors [43]. The dense packing and high vascularity of the CC might partially explain its particular vulnerability to neuro-inflammation and demyelination [53], potentially induced by chemotherapy exposure.

Within the chemotherapy subgroup, the lower FDC was less pronounced when mothers received anthracyclines, indicating that these anthracycline-based combination treatments might generally provide a lower risk for affecting the developing brain.

Interestingly, in the whole study group a higher FCS was observed in the left prefrontal subcortical WM, with similarly a higher FDC being observed in the chemotherapy subgroup in the right anterior thalamic radiation. Both findings indicate stronger structural connectivity in the anterior thalamic radiations compared to controls. Seeing the importance of this WM tract for executive functioning [54], which was found earlier to be affected in both this population [15] and cancer survivors [55], [56], [57], the increase in FDC/FCS in this structure might result from a neuroplastic compensatory mechanism. However, this might also reflect altered WM developmental patterns due to changes in the pre- and postnatal environment, both directly and indirectly linked to the maternal cancer.

Prematurity in this study only showed a significant impact on the mean curvature in the left posterior cingulate, resonating with earlier findings of altered functional connectivity with prematurity in this region [58], as well as with earlier findings on the discrepancy between in utero and postnatal gyrification [59]. On the other hand, the observed limited effect of GA confirms earlier findings that neurocognitive effects of prenatal exposure to cancer and its therapy are not limited to the frequent occurrence of preterm birth [14,15].

Some limitations of this study should be mentioned. Due to the rare incidence of cancer during pregnancy, data acquisition spanned a five-year period. The effects of scanner variability during this period were limited by keeping the scanner set-up and protocol unchanged over the whole period and by simultaneously recruiting study and control group children. Next, while the total sample size allows for observations on overall group effects, the heterogeneity of this population necessitates caution in the interpretation of the results for specific therapies during pregnancy. Furthermore, only 12/42 mothers in the study group did not receive chemotherapy during pregnancy, and this group of patients is often diagnosed later in pregnancy or with less aggressive tumours, making it especially difficult to distinguish effects of therapy from other cancer-related mechanisms. Moreover, when interpreting these findings, we should note that this study only included Belgian patients, treated between 2005 and 2010. Indeed, this study cannot account for international differences in treatment and more recent advances in clinical more. On the other hand, most treatments women received in this cohort still adhere to today's guidelines [4,[60], [61], [62]]. Furthermore, non-cancer-related heterogeneity was limited by testing children within a small age range and effects of prematurity were controlled for by matching controls on GA on a 1:1 ratio. Finally, no data on maternal stress and anxiety were available for these children, making it difficult to discriminate between prenatal stress-related or cancer-related impact on neurodevelopment. However, current findings do not align with previous findings on the effects of prenatal stress on neurodevelopment [63,64], making it an unlikely pathway to completely explain the discrepancies in brain development observed in this study.

This study observed local structural WM and GM differences, but no whole-brain or functional differences, in children born after cancer-complicated pregnancies compared to matched controls. Platinum derivatives during pregnancy were indicated as a potential risk factor for decreased cortical gyrification of the left superior temporal gyrus. However, chemotherapy during pregnancy in general did not significantly contribute to these findings, resonating with earlier findings [14,19] describing the role of the psychosocial impact of cancer during pregnancy on the neurocognitive functioning of the child. Balancing between pros and cons on the use of chemotherapy during pregnancy, the current data favour the use of chemotherapy during pregnancy when clinically indicated.

## Data sharing statement

The anonymized data that support the findings of this study, as well as related documents, are available from the corresponding author upon reasonable request.

## Author contribution statement

- Jeroen Blommaert: data collection (MRI), management (MRI), analysis and interpretation; methodology; writing (original draft, review and editing); project financing
- Ahmed Radwan: data analysis and interpretation; methodology; writing (review and editing)
- Charlotte Sleurs: data collection (MRI) and interpretation; methodology; writing (review and editing)
- Charlotte Maggen: data collection (Clinical), management (Clinical) and interpretation; methoodology; writing (review and editing)
- Mathilde van Gerwen: data management (neuropsychological) and interpretation; methodology; writing (review and editing)
- Vera Wolters: data interpretation; writing (review and editing)
- Daan Christiaens: data interpretation; methodology; writing (review and editing)
- Ronald Peeters: MR protocol design; MR system management; writing (review and editing)
- Patrick Dupont: methodology; writing (review and editing)
- Stefan Sunaert: MR protocol design; data interpretation; methodology; writing (review and editing)
- Kristel van Calsteren: Study conceptualization and design; data interpretation; writing (review and editing)
- Sabine Deprez: Study conceptualization and design; data interpretation; methodology; writing (review and editing)
- Frédéric Amant: Study conceptualization and design; data interpretation; writing (review and editing); project financing

S.D. and F.A. equally contributed to the manuscript

## Declaration of Competing Interest

All authors report no disclosures.

## Appendix Supplementary materials

Image, application 1

Image, application 2
