# Supplementary material for: The impact of cancer and chemotherapy during pregnancy on child neurodevelopment: A multimodal neuroimaging analysis
Source: eClinicalMedicine. 2020 Oct 21;28:100598. doi: 10.1016/j.eclinm.2020.100598 (PMC7700909; doi:10.1016/j.eclinm.2020.100598)
Supplement: Supplementary file 1 [file mmc1.pdf]

1. Supplementary Materials

1. Supplementary Materials **1**

1.1. Supplementary Figures *2*

1.2. Supplementary Tables *3*

1.3. Data analysis *3*

1.3.1. Cortical and subcortical morphometry 4

1.3.2. White matter microstructure 5

1.3.3. Functional connectivity 7

1.3.4. whole-brain connectomics 8

1.4. Neurological abnormalities *8*

1.5. References *9*

1.1. Supplementary Figures

Supplementary figure 1 Observed differences in WM microstructure at trend level. Trends for the effects in **Error! Reference source not found.** are visualized at $p_{FWE}<.1$, using connectivity-based fixel enhancement[1] with family-wise-error correction. All results are rendered with 200000 streamlines (as described earlier[2]), mapped on 10mm interval slices and coloured with conventional directional colour encoding. FCS: fibre cross-section. FDC: Fibre density and cross-section.

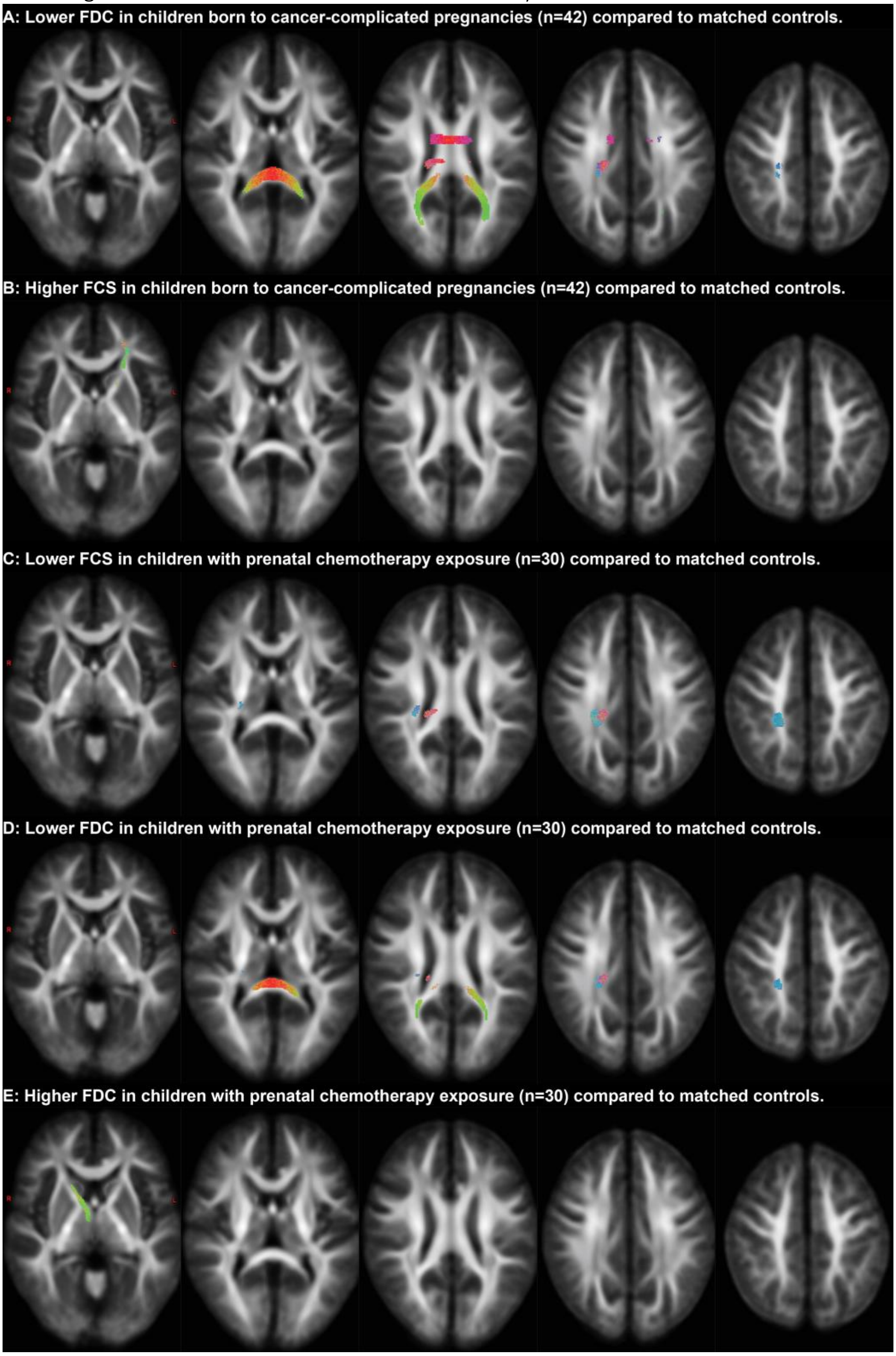

1.2. SUPPLEMENTARY TABLES

Supplementary table 1: Image quality metrics. All metrics are calculated on the groups after exclusion criteria were applied: n=42 per group for rs-fMRI and diffusion images and n=38 per group for T1 images. rs-fMRI: resting-state functional MRI. Q1: first quartile. Q3: third quartile.

	Study Group			Control group		
	Median	Q1	Q2	Median	Q2	Q3
**T1-weighted**						
Contrast-to-noise ratio	5.25	5.07	5.42	5.18	5.04	5.44
Coefficient of joint variation	0.230	0.221	0.241	0.229	0.201	0.238
Entropy focus criterion	0.604	0.586	0.671	0.622	0.562	0.739
**rs-fMRI**						
Mean framewise displacement (mm)	0.215	0.164	0.275	0.197	0.164	0.264
Temporal signal-to-noise ratio	61.6	51.7	74.4	60.4	49.7	76.0
**Diffusion**						
Mean translation between slices (mm)	0.302	0.273	0.350	0.297	0.278	0.342
Mean rotation between slices (degrees)	0.243	0.221	0.294	0.248	0.220	0.283
Outlier ratio	0.079	0.058	0.100	0.076	0.060	0.092

Supplementary table 2: Global brain volumes. Brain volumes are expressed in ml. Significance is assessed at p<.05.

Study group	Parameter	Study group		Control group		p		
		Mean	SD	Mean	SD	Group	GA	Group by GA
All cancers (n=38)	Total brain volume	1205	113	1239	85	*.143*	*.898*	*.666*
All cancers (n=38)	Grey matter	732	68	755	49	*.090*	*.650*	*.947*
All cancers (n=38)	White matter	473	48	483	41	*.298*	*.751*	*.374*
Chemo (n=27)	Total brain volume	1208	123	1238	90	*.315*	*.467*	*.933*
Chemo (n=27)	Grey matter	737	73	754	51	*.310*	*.836*	*.744*
Chemo (n=27)	White matter	471	52	483	43	*.355*	*.167*	*.526*

1.3. DATA ANALYSIS

All images were processed by combining publicly available toolboxes and in-house developed scripts (using bash and Matlab v2018b). Unless otherwise mentioned, standard settings were used. Prior to processing, all images were converted to the Brain Imaging Data Structure (BIDS)[3] and visually inspected for issues. Quantitative quality parameters of the T1-weighted images and rs-fmri were calculated using MRIQC (v0.14.2)[4] and using shardrecon[5] for the diffusion-weighted images. For the T1-weighted images, the following quality

parameters are reported: contrast-to-noise ratio (CNR)[6], coefficient of joint variation (CJV)[7] and entropy focus criterion (EFC)[8]. For rs-fMRI mean framewise displacement (FD)[9] and temporal signal-to-noise ratio (tSNR)[10] are reported. For diffusion images mean Euclidean framewise translation and rotation, as well as the outlier ratio (OR) were used, all using the estimates from the SHARD-recon motion correction[5]. The OR was defined as $1 - \frac{\sum slice\ weights}{Total\ number\ of\ slices}$. Image quality metrics are provided in Supplementary table 1. Further visual checks of data quality were performed at multiple steps of the data processing.

1.3.1. Cortical and subcortical morphometry

Cortical and subcortical grey matter features were extracted from the T1-weighted images using Mindboggle (v1.3.8)[11]. This analysis hybridizes parcellations of the Freesurfer (v6.0.0)[12] and ANTs (v2.3.1) cortical thickness[13,14] pipelines. While this method corrects for underestimation of GM on the outside of the brain, it might be prone to misclassification of dura matter as GM. For this reason, the standard brain mask in Freesurfer was replaced in this analysis by a mask generated by HD-BET[15]. Similarly, this brain mask was applied to the ANTs segmentation. N4 biasfield correction (ANTs v2.3.1)[13] was applied on the initial T1-weighted image prior to Freesurfer parcellation.

Images scoring in the worst 20 percentile of CJV,CNR or EFC (n=30) were visually rated by a trained radiologist (AR) into bad (n=1), doubtful (n=16) or good (n=23) quality[16]. Images of initially doubtful quality were in-depth re-assessed after surface reconstruction was performed, those with initial bad quality (n=1), were rejected. For each image with doubtful quality, parcellated images were again rated for errors by the same neuroradiologist (AR), and either accepted or rejected for morphometrical analysis. All other surface reconstructions and parcellations were evaluated for major issues, using 4mm interleaved axial slices. Participants

who were matched to children excluded from the morphometrical analysis, were re-matched to one another when meeting the matching criteria. All reconstructions were deemed of sufficient quality for use in the connectome analyses (including those excluded from the morphometrical analysis).

The corrected Freesurfer Desikan-Killiany parcellations [17,18] were used for region-based statistical analysis. GM (subcortical+cortical), WM and total brain (GM+WM) volumes were compared between groups, as well as the volume of the different subcortical structures[18]. Different mean cortical features[11] of each region were investigated: mean thickness (Freesurfer), surface area, mean travel depth and mean curvature. Statistical analysis was performed within Matlab, using the 'anovan' function. The Benjamini-Hochberg procedure was used to control for multiple comparisons of cortical features in 62 regions and subcortical volumes in 16 regions. For subcortical volume analyses, total brain volume was included as a covariate.

1.3.2. White matter microstructure

White matter microstructure was examined using a fixel-based analysis on the multi-shell diffusion-weighted images, which was performed using MRtrix[19] (v3.0). Pre-processing consisted of the following steps: denoising[20], Gibbs ringing removal[21], distortion correction[22] (FSL v5.0.11 topup, between b0 and reversed phase image, using prior AP padding with 30 voxels), motion correction[5] (SHARD-recon, SH basis per shell=0,4,6, reduced basis=2,0, reg=0.005, zreg=0.001), biasfield correction (ANTs v2.3.1 N4 correction[13], shrink factor 1, mesh

resolution=150mm, spline order 3, maximum iterations=500x500, convergence threshold=0.0) and upsampling to an isotropic voxel size of 1.3mm.

Group-average response functions were calculated for WM, GM and CSF. Next, fibre orientation distributions (FOD) were calculated in each voxel using constrained spherical deconvolution (CSD) and Joint bias field correction and intensity normalization was performed. All FOD images were registered to an unbiased population template, which was calculated using data from 15 randomly selected children from both study and control group. Fixel-based parameters of fibre density (FD), fibre cross-section (FCS) and their combined metric of fibre density and cross-section (FDC) were calculated as described elsewhere[2].

Whole-brain tractography was performed on the template FOD (iFOD2,20 million fibres, maximum angle=22.5, minimum/maximum length=10/250mm, power=1.0, FOD cut-off=.06) and reduced to 2 million fibres using Spherical-deconvolution informed filtering of tractograms (SIFT)[23]. Differences in FD, FCS (logarithmically scaled) and FDC were assessed at each individual fixel using connectivity-based fixel enhancement[1] (CFE). Only fixels where more than 150 streamlines pass through were included in the final analysis. This resulted in the final analysis mask containing 316424 fixels (61% of the original fixel mask). ICV was calculated by back-transforming the group mask to subject-space, as described earlier by Pannek et al.[24]. normalized ICV was used as a covariate in analyses of FCS and FDC[25]. Significance was inferred at $p<.05$, corrected for multiple corrections using family-wise error correction within the CFE framework[1].

Structural connectomes were generated for each participant. First, the T1-weighted image and corrected Freesurfer parcellation[11,17] of each participant were registered to first volume of the normalized WM FOD images (ANTs rigid Inter modality Intra subject registration). Next,

Whole-brain tractography was performed on normalized WM FOD images (iFOD2, 10 million fibres, minimum/maximum length=5/300mm, dynamic seeding), using anatomically constrained tractography (ACT, based on the parcellations, including amygdalae and hippocampi as subcortical GM) and SIFT2 re-weighting of streamlines[26]. Finally, weighted undirected connectomes were generated based on SIFT2 re-weighted streamlines and corrected Freesurfer parcellation, with edge values normalized to the maximum edge weight.

1.3.3. Functional connectivity

All rs-fMR images were pre-processed using fmriprep (v20.0.1)[27], with following non-standard settings: no Freesurfer surface reconstruction, using a fieldmap-less approach for correcting EPI distortion artefacts[28], calculating motion confounds using ICA-AROMA[29] (aggressive setting) and using the anatomical image as output space. Next, nuisance regression and temporal band pass filtering (.009-.1Hz) was applied using the denoiser toolbox (https://github.com/arielletambini/denoiser). This nuisance regression was applied using the following parameters from the fmriprep output: CSF (1 parameter) and WM (1 parameter) signal, framewise translations (3 parameters) and (3 parameters) rotations, ICA-AROMA motion confounds (variable number of parameters) and signal drift (6 parameters). Further analysis was conducted using the CONN toolbox (v19.b)[30]. Within this toolbox, images were segmented (GM, WM and CSF) and normalized to MNI. Next, a Gaussian smoothing kernel of 6mm isotropic was applied to the functional images. Based on the a priori hypothesis of possible impact of chemotherapy on executive functioning, nodes of the default mode (DMN), fronto-parietal (FPN), dorsal attention (DAN) and salience networks (SN) were selected from the CONN (v19.b)[30] network atlas. This resulted in a total of 17 regions included in this analysis. ROI-to-ROI functional coherence between these regions was estimated using bivariate

correlations. Significance was assessed at $p<.05$, false discovery rate corrected for comparing 171 connections.

Undirected weighted functional connectomes were generated using absolute bivariate correlations between regions of the corrected Freesurfer parcellation[11,17] in the mean timeseries of the pre-processed functional images (upsampled to match the anatomical image).

1.3.4. WHOLE-BRAIN CONNECTOMICS

Graph theory analysis was performed using in-house developed MATLAB scripts and the Brain Connectivity toolbox (v2019-03-03). Self-connections were removed from all connectomes. Whole-brain graph theory measures of characteristic path length, global and local efficiency and clustering coefficient[31] were calculated. Characteristic path length and global efficiency were calculated using Dijkstra's algorithm, with the connection-length matrix defined by the inverse edge weights (except for self-connections which have a zero length). Clustering coefficient and local efficiency measures were calculated as recommended by Wang et al.[31] For each connectome, 100 random graphs were calculated by randomly permuting the edges, while keeping the connectome symmetry, the zero-weight of the self-connections and excluding graphs with disconnected nodes. Normalized graph measures were calculated by dividing the original graph measures of the connectome by the mean of the graph measures of equivalent random graphs. Statistical comparison of graph measures was performed using standard parametric testing, using SPSS (v19.0) univariate GLM. A Bonferroni correction was introduced for comparing 2 types of graphs. No correction of multiple comparisons was introduced for the number of global graph measures as these are highly interdependent.

1.4. NEUROLOGICAL ABNORMALITIES

Four children presented neurological abnormalities on the MRI, as rated by a clinical neuroradiologist, and had to be excluded from this study. Two children were born to a mother with cancer during pregnancy, with both mothers receiving chemotherapy during pregnancy. The first child, a girl born at 31 weeks of gestation, presented a white matter lesion at the head of right caudate, which could be traced back to a perinatal subependymal bleeding. However, as the child was born at a GA of 31 weeks, and this is a common pathology in preterm children, this cannot be directly related to the maternal cancer treatment. The second child, a boy born at 38 weeks of gestation, was diagnosed with a focal cortical dysplasia in the left medial parieto-occipital sulcus. This is a pathology of neuronal migration, which most likely is due to a fetal impact at a GA of 3-5 months[32]. However, as the first chemotherapy cycle was administered at a GA of 29 weeks a link between this therapy and the development of the focal cortical dysplasia is unlikely.

In the control group incidental findings were observed in two children. One girl, born at a GA of 36 weeks, presented bilateral parietal and frontal periventricular leukomalacia. A second girl, born at a GA of 39 weeks, had a left temporo-insular subependymal cyst.

1.5. References

1 Raffelt DA, Smith RE, Ridgway GR, *et al.* Connectivity-based fixel enhancement: Whole-brain statistical analysis of diffusion MRI measures in the presence of crossing fibres. *Neuroimage* 2015; **117**: 40–55.

2 Raffelt DA, Tournier JD, Smith RE, *et al.* Investigating white matter fibre density and morphology using fixel-based analysis. *Neuroimage* 2017; **144**: 58–73.

3 Gorgolewski KJ, Auer T, Calhoun VD, *et al.* The brain imaging data structure, a format for

organizing and describing outputs of neuroimaging experiments. *Sci Data* 2016; **3**: 160044.

4 Esteban O, Birman D, Schaer M, Koyejo OO, Poldrack RA, Gorgolewski KJ. MRIQC: Advancing the automatic prediction of image quality in MRI from unseen sites. *PLoS One* 2017; **12**: e0184661.

5 Christiaens D, Cordero-Grande L, Pietsch M, *et al.* Scattered slice SHARD reconstruction for motion correction in multi-shell diffusion MRI of the neonatal brain. 2019; published online May 8. http://arxiv.org/abs/1905.02996.

6 Magnotta VA, Friedman L. Measurement of Signal-to-Noise and Contrast-to-Noise in the fBIRN Multicenter Imaging Study. *J Digit Imaging* 2006; **19**: 140–7.

7 Ganzetti M, Wenderoth N, Mantini D. Intensity Inhomogeneity Correction of Structural MR Images: A Data-Driven Approach to Define Input Algorithm Parameters. *Front Neuroinform* 2016; **10**: 10.

8 Atkinson D, Hill DLG, Stoyle PNR, Summers PE, Keevil SF. Automatic correction of motion artifacts in magnetic resonance images using an entropy focus criterion. *IEEE Trans Med Imaging* 1997; **16**: 903–10.

9 Power JD, Barnes KA, Snyder AZ, Schlaggar BL, Petersen SE. Spurious but systematic correlations in functional connectivity MRI networks arise from subject motion. *Neuroimage* 2012; **59**: 2142–54.

10 Krüger G, Glover GH. Physiological noise in oxygenation-sensitive magnetic resonance imaging. *Magn Reson Med* 2001; **46**: 631–7.

11 Klein A, Ghosh SS, Bao FS, *et al.* Mindboggling morphometry of human brains. 2017 DOI:10.1371/journal.pcbi.1005350.

12 Fischl B. FreeSurfer. *Neuroimage* 2012; **62**: 774–81.

13 Tustison NJ, Avants BB, Cook PA, *et al.* N4ITK: Improved N3 Bias Correction. *IEEE Trans Med Imaging* 2010; **29**: 1310–20.

14 Tustison NJ, Cook PA, Klein A, *et al.* Large-scale evaluation of ANTs and FreeSurfer cortical thickness measurements. *Neuroimage* 2014; **99**: 166–79.

15 Isensee F, Schell M, Pflueger I, *et al.* Automated brain extraction of multisequence MRI using artificial neural networks. *Hum Brain Mapp* 2019; **40**: 4952–64.

16 Backhausen LL, Herting MM, Buse J, Roessner V, Smolka MN, Vetter NC. Quality Control of Structural MRI Images Applied Using FreeSurfer—A Hands-On Workflow to Rate Motion Artifacts. *Front Neurosci* 2016; **10**: 558.

17 Desikan RS, Ségonne F, Fischl B, *et al.* An automated labeling system for subdividing the human cerebral cortex on MRI scans into gyral based regions of interest. *Neuroimage* 2006; **31**: 968–80.

18 Fischl B, Salat DH, van der Kouwe AJW, *et al.* Sequence-independent segmentation of magnetic resonance images. *Neuroimage* 2004; **23**: S69–84.

19 Tournier J-D, Smith RE, Raffelt DA, *et al.* MRtrix3: A fast, flexible and open software framework for medical image processing and visualisation. *bioRxiv* 2019. DOI:10.1101/551739.

20 Veraart J, Novikov DS, Christiaens D, Ades-aron B, Sijbers J, Fieremans E. Denoising of

diffusion MRI using random matrix theory. *Neuroimage* 2016; **142**: 394–406.

21 Kellner E, Dhital B, Kiselev VG, Reisert M. Gibbs-ringing artifact removal based on local subvoxel-shifts. *Magn Reson Med* 2016; **76**: 1574–81.

22 Andersson JLR, Skare S, Ashburner J. How to correct susceptibility distortions in spin-echo echo-planar images: application to diffusion tensor imaging. *Neuroimage* 2003; **20**: 870–88.

23 Smith RE, Tournier J-D, Calamante F, Connelly A. SIFT: Spherical-deconvolution informed filtering of tractograms. *Neuroimage* 2013; **67**: 298–312.

24 Pannek K, Fripp J, George JM, *et al.* Fixel-based analysis reveals alterations is brain microstructure and macrostructure of preterm-born infants at term equivalent age. *NeuroImage Clin* 2018; **18**: 51–9.

25 Smith RE, Dhollander T, Connelly A. On the regression of intracranial volume in Fixel-Based Analysis Discussion / Conclusion. In: Proceedings of the International Society for Magnetic Resonance in Medicine. 2019: 3385.

26 Smith RE, Tournier J-D, Calamante F, Connelly A. SIFT2: Enabling dense quantitative assessment of brain white matter connectivity using streamlines tractography. *Neuroimage* 2015; **119**: 338–51.

27 Esteban O, Markiewicz CJ, Blair RW, *et al.* fMRIPrep: a robust preprocessing pipeline for functional MRI. *Nat Methods* 2019; **16**: 111–6.

28 Wang S, Peterson DJ, Gatenby JC, Li W, Grabowski TJ, Madhyastha TM. Evaluation of Field Map and Nonlinear Registration Methods for Correction of Susceptibility Artifacts

in Diffusion MRI. *Front Neuroinform* 2017; **11**: 17.

29 Pruim RHR, Mennes M, van Rooij D, Llera A, Buitelaar JK, Beckmann CF. ICA-AROMA: A robust ICA-based strategy for removing motion artifacts from fMRI data. *Neuroimage* 2015; **112**: 267–77.

30 Whitfield-Gabrieli S, Nieto-Castanon A. Conn : A Functional Connectivity Toolbox for Correlated and Anticorrelated Brain Networks. *Brain Connect* 2012; **2**: 125–41.

31 Wang Y, Ghumare E, Vandenberghe R, Dupont P. Comparison of Different Generalizations of Clustering Coefficient and Local Efficiency for Weighted Undirected Graphs. *Neural Comput* 2017; **29**: 313–31.

32 Jansen AC, Keymolen K. Fetal and neonatal neurogenetics, 1st edn. Elsevier B.V., 2019 DOI:10.1016/B978-0-444-64029-1.00005-9.
