# Supplementary material for: The impact of cancer and chemotherapy during pregnancy on child neurodevelopment: A multimodal neuroimaging analysis
Source: eClinicalMedicine. 2020 Oct 21;28:100598. doi: 10.1016/j.eclinm.2020.100598 (PMC7700909; doi:10.1016/j.eclinm.2020.100598)
Supplement: Supplementary file 2 [file mmc2.docx]

STROBE Statement—checklist of items that should be included in reports of observational studies

|  | Item No. | Recommendation | Page  No. | Relevant text from manuscript |
| --- | --- | --- | --- | --- |
| **Title and abstract** | 1 | (*a*) Indicate the study’s design with a commonly used term in the title or the abstract | 2 | “In this cohort study,” |
|  |  | (*b*) Provide in the abstract an informative and balanced summary of what was done and what was found | 2 | Methods and findings section |
| Introduction | | | |  |
| Background/rationale | 2 | Explain the scientific background and rationale for the investigation being reported | 4-5 | Section 1 |
| Objectives | 3 | State specific objectives, including any prespecified hypotheses | 5 | “increase understanding of the potentially detrimental effects of cancer and its treatment during pregnancy on structural and functional brain development in the offspring at nine years old.” |
| Methods | | | |  |
| Study design | 4 | Present key elements of study design early in the paper | 5-6 | First paragraph of section 2.1 |
| Setting | 5 | Describe the setting, locations, and relevant dates, including periods of recruitment, exposure, follow-up, and data collection | 6 | “Children in the study group were born to mothers with a cancer diagnosis during pregnancy.” “All children were tested between 2015 and 2020 at the age of nine years, at the university hospital of Leuven, Belgium “ |
| Participants | 6 | (*a*) *Cohort study*—Give the eligibility criteria, and the sources and methods of selection of participants. Describe methods of follow-up  *Case-control study*—Give the eligibility criteria, and the sources and methods of case ascertainment and control selection. Give the rationale for the choice of cases and controls  *Cross-sectional study*—Give the eligibility criteria, and the sources and methods of selection of participants | 5-6 | Section 2.1 paragraph 1 |
|  |  | (*b*) *Cohort study*—For matched studies, give matching criteria and number of exposed and unexposed  *Case-control study*—For matched studies, give matching criteria and the number of controls per case | 6 | “Children in the control group, born to healthy mothers, were matched on a 1:1 ratio regarding gestational age at birth (GA, maximum 1 week difference), age (9 years old) and sex” |
| Variables | 7 | Clearly define all outcomes, exposures, predictors, potential confounders, and effect modifiers. Give diagnostic criteria, if applicable | 7-9 | Sections 2.3 and 2.4 |
| Data sources/ measurement | 8* | For each variable of interest, give sources of data and details of methods of assessment (measurement). Describe comparability of assessment methods if there is more than one group | 6-7 | Section 2.1 paragraph 2 and 3.  And section 2.2 |
| Bias | 9 | Describe any efforts to address potential sources of bias | 7,15 | “All analyses included multiple steps of bias, motion and artefact correction54–60, as well as visual and quantitative quality assurance60,61.” “The effects of scanner variability during this period were limited by keeping the scanner set-up and protocol unchanged over the whole period and by simultaneously recruiting study and control group children.” |
| Study size | 10 | Explain how the study size was arrived at | 9-10 | Section 3.1, paragraph 1 and figure 1 |

Continued on next page

| Quantitative variables | 11 | Explain how quantitative variables were handled in the analyses. If applicable, describe which groupings were chosen and why | 8-9 | Section 2.4 |
| --- | --- | --- | --- | --- |
| Statistical methods | 12 | (*a*) Describe all statistical methods, including those used to control for confounding | 8-9 | Section 2.4 |
|  |  | (*b*) Describe any methods used to examine subgroups and interactions | 8-9 | Section 2.4 |
|  |  | (*c*) Explain how missing data were addressed | 38 | “Participants who were matched to children excluded from the morphometrical analysis, were re-matched to one another when meeting the matching criteria.”, Figure 1 |
|  |  | (*d*) *Cohort study*—If applicable, explain how loss to follow-up was addressed  *Case-control study*—If applicable, explain how matching of cases and controls was addressed  *Cross-sectional study*—If applicable, describe analytical methods taking account of sampling strategy |  |  |
|  |  | (*e*) Describe any sensitivity analyses | 9 | “exploratory analyses were performed within SPSS (v19.0) investigating the association between brain functioning/structure, psycho-behavioural functioning and obstetrical/oncological outcome, using spearman correlations for numerical variables and Mann-Whitney (MW) U-test for categorical variables." |
| Results | | | | |
| Participants | 13* | (a) Report numbers of individuals at each stage of study—eg numbers potentially eligible, examined for eligibility, confirmed eligible, included in the study, completing follow-up, and analysed | Figure 1 | Figure 1 |
|  |  | (b) Give reasons for non-participation at each stage | Figure 1 | Figure 1 |
|  |  | (c) Consider use of a flow diagram | Figure 1 | Figure 1 |
| Descriptive data | 14* | (a) Give characteristics of study participants (eg demographic, clinical, social) and information on exposures and potential confounders | Table 1 | Table 1 |
|  |  | (b) Indicate number of participants with missing data for each variable of interest | Figure 1 | Figure 1 |
|  |  | (c) *Cohort study*—Summarise follow-up time (eg, average and total amount) | Table 1 | Table 1 |
| Outcome data | 15* | *Cohort study*—Report numbers of outcome events or summary measures over time | *8* | *Section 2.3* |
|  |  | *Case-control study—*Report numbers in each exposure category, or summary measures of exposure |  |  |
|  |  | *Cross-sectional study—*Report numbers of outcome events or summary measures |  |  |
| Main results | 16 | (*a*) Give unadjusted estimates and, if applicable, confounder-adjusted estimates and their precision (eg, 95% confidence interval). Make clear which confounders were adjusted for and why they were included |  |  |
|  |  | (*b*) Report category boundaries when continuous variables were categorized | Table 1 | “Very preterm children have GA 28-32 weeks, moderate to late preterm children have GA 32-37 weeks.” |
|  |  | (*c*) If relevant, consider translating estimates of relative risk into absolute risk for a meaningful time period | NA | NA |

Continued on next page

| Other analyses | 17 | Report other analyses done—eg analyses of subgroups and interactions, and sensitivity analyses | 8-9 | Section 2.4 |
| --- | --- | --- | --- | --- |
| Discussion | | | | |
| Key results | 18 | Summarise key results with reference to study objectives | 15 | “This study observed local structural WM and GM differences, but no whole-brain or functional differences, in children born after cancer-complicated pregnancies compared to matched controls. Platinum derivatives during pregnancy were indicated as a potential risk factor for decreased cortical gyrification of the left superior temporal gyrus” |
| Limitations | 19 | Discuss limitations of the study, taking into account sources of potential bias or imprecision. Discuss both direction and magnitude of any potential bias | 15 | “Some limitations of this study should be mentioned …. “ |
| Interpretation | 20 | Give a cautious overall interpretation of results considering objectives, limitations, multiplicity of analyses, results from similar studies, and other relevant evidence | 16 | “Balancing between pros and cons on the use of chemotherapy during pregnancy, the current data favour the use of chemotherapy during pregnancy when clinically indicated.” |
| Generalisability | 21 | Discuss the generalisability (external validity) of the study results | 15 | “while the total sample size allows for observations on overall group effects, the heterogeneity of this population necessitates caution in the interpretation of the results for specific therapies during pregnancy” |
| Other information | |  | | |
| Funding | 22 | Give the source of funding and the role of the funders for the present study and, if applicable, for the original study on which the present article is based | 9,17 | “The funding sources had no role in writing of the manuscript or the decision to submit it for publication”, section 8 |

*Give information separately for cases and controls in case-control studies and, if applicable, for exposed and unexposed groups in cohort and cross-sectional studies.

**Note:** An Explanation and Elaboration article discusses each checklist item and gives methodological background and published examples of transparent reporting. The STROBE checklist is best used in conjunction with this article (freely available on the Web sites of PLoS Medicine at http://www.plosmedicine.org/, Annals of Internal Medicine at http://www.annals.org/, and Epidemiology at http://www.epidem.com/). Information on the STROBE Initiative is available at [www.strobe-statement.org](http://www.strobe-statement.org).

**Supplementary table 1: Image quality metrics.** All metrics are calculated on the groups after exclusion criteria were applied: n=42 per group for rs-fMRI and diffusion images and n=38 per group for T1 images. ﻿rs-fMRI: resting-state functional MRI. Q1: first quartile. Q3: third quartile.

|  | **Study Group** | | | **Control group** | | |
| --- | --- | --- | --- | --- | --- | --- |
|  | Median | Q1 | Q2 | Median | Q2 | Q3 |
| **T1-weighted** |  |  |  |  |  |  |
| Contrast-to-noise ratio | 5.25 | 5.07 | 5.42 | 5.18 | 5.04 | 5.44 |
| Coefficient of joint variation | 0.230 | 0.221 | 0.241 | 0.229 | 0.201 | 0.238 |
| Entropy focus criterion | 0.604 | 0.586 | 0.671 | 0.622 | 0.562 | 0.739 |
| **rs-fMRI** |  |  |  |  |  |  |
| Mean framewise displacement (mm) | 0.215 | 0.164 | 0.275 | 0.197 | 0.164 | 0.264 |
| Temporal signal-to-noise ratio | 61.6 | 51.7 | 74.4 | 60.4 | 49.7 | 76.0 |
| **Diffusion** |  |  |  |  |  |  |
| Mean translation between slices (mm) | 0.302 | 0.273 | 0.350 | 0.297 | 0.278 | 0.342 |
| Mean rotation between slices (degrees) | 0.243 | 0.221 | 0.294 | 0.248 | 0.220 | 0.283 |
| Outlier ratio | 0.079 | 0.058 | 0.100 | 0.076 | 0.060 | 0.092 |

**Supplementary table 2: Global brain volumes.** Brain volumes are expressed in ml. Significance is assessed at p<.05.

| **Study group** | **Parameter** | **Study group** | | **Control group** | | **P_FDR_** | | |
| --- | --- | --- | --- | --- | --- | --- | --- | --- |
|  |  | Mean | SD | Mean | SD | Group | GA | Group by GA |
| All cancers (n=38) | Total brain volume | 1205 | 113 | 1239 | 85 | *.143* | *.898* | *.666* |
| All cancers (n=38) | Grey matter | 732 | 68 | 755 | 49 | *.090* | *.650* | *.947* |
| All cancers (n=38) | White matter | 473 | 48 | 483 | 41 | *.298* | *.751* | *.374* |
| Chemo (n=27) | Total brain volume | 1208 | 123 | 1238 | 90 | *.315* | *.467* | *.933* |
| Chemo (n=27) | Grey matter | 737 | 73 | 754 | 51 | *.310* | *.836* | *.744* |
| Chemo (n=27) | White matter | 471 | 52 | 483 | 43 | *.355* | *.167* | *.526* |
